# Lattice Boltzmann Simulation of Shale Gas Transport in Organic Nano-Pores

**DOI:** 10.1038/srep04843

**Published:** 2014-05-02

**Authors:** Xiaoling Zhang, Lizhi Xiao, Xiaowen Shan, Long Guo

**Affiliations:** 1 State Key Laboratory of Petroleum Resources and Prospecting, China University of Petroleum, Beijing 102249, People's Republic of China; 2 Beijing Aeronautical Science & Technology Research Institute of COMAC (BASTRI), People's Republic of China

## Abstract

Permeability is a key parameter for investigating the flow ability of sedimentary rocks. The conventional model for calculating permeability is derived from Darcy's law, which is valid only for continuum flow in porous rocks. We discussed the feasibility of simulating methane transport characteristics in the organic nano-pores of shale through the Lattice Boltzmann method (LBM). As a first attempt, the effects of high Knudsen number and the associated slip flow are considered, whereas the effect of adsorption in the capillary tube is left for future work. Simulation results show that at small Knudsen number, LBM results agree well with Poiseuille's law, and flow rate (flow capacity) is proportional to the square of the pore scale. At higher Knudsen numbers, the relaxation time needs to be corrected. In addition, velocity increases as the slip effect causes non negligible velocities on the pore wall, thereby enhancing the flow rate inside the pore, i.e., the permeability. Therefore, the LBM simulation of gas flow characteristics in organic nano-pores provides an effective way of evaluating the permeability of gas-bearing shale.

The production of shale gas largely depends on the flow ability of natural gas, which is affected by several inherent characteristics of gas shale, such as gas composition, organic richness, the geometry structure of the nano-pores, bedding and micro fractures, and more fundamentally, the deviation of the gas flow from the description of continuum fluid mechanics because of the considerable effects of high Knudsen numbers. Kerogen pores are micro-pores with sizes ranging from 2 nm to 50 nm. Although some larger pores may exist, the average kerogen pore size is typically below 10 nm^1^,^2^. Being the ratio of the molecular mean free path and the characteristic length, the Knudsen number *Kn* is used to define fluid flow regime, i.e., continuum flow (*Kn*<10^−3^), slip flow (10^−3^<*Kn*<10^−1^), transitional flow (0.1<*Kn*<10), and free molecular flow (10<*Kn*). For normal temperature (300 K to 360 K) and pressure (5 MPa to 28 MPa) conditions in shale gas plays, the molecular mean free path of methane may be between 0.3 and 2.2 nm. *Kn* is 10^−2^ to 10^0^ in the 2 nm to 50 nm range of shale pores; thus, the continuum assumption of fluid flow may break down, and Navier–Stokes (N–S) equations with no-slip boundaries may be invalid^3^. Normal numerical simulation based on N–S equations loses the basis to correctly predict the flow characteristics of shale gas. An empirical and simplified permeability model used for conventional reservoir studies cannot be used to estimate the permeability of gas shale.

To analyze gas flow in nano-pores, various analytical and numerical methods have been developed. First, in the molecular dynamics (MD) method, fluid is treated as a group of discrete particles and molecular models because the temperature-sensitive kinetic distribution of all independent particles is often used^4^. Although MD is theoretically suitable for simulating interaction in small-scale pores, it is not practical for simulating the nano-scale pore networks of shale because of the very high computational cost. Most MD calculations are limited to 10^−15^ s' time step, and results are limited to a very short time scale of ps or ns. High computing power and storage capacity of computers are needed. Consequently, the MD method can be used to simulate only few molecular systems rather than the complex flow field. By contrast, macro-level simulation methods mainly use finite element, finite difference, finite volume, or spectral methods to discretize N–S equations and to obtain the numerical solution based on the continuous fluid flow hypothesis^3^,^5^,^6^. DSMC can generally be used to simulate rarefied gas flows, mostly in outer space applications. However, it's very expensive computationally and highly noisy at small Mach numbers^7^. In addition these methods have great difficulties in simulating fluid systems with mixed fluid phase interfaces and are therefore generally unsuitable for the complex flow problem of shale gas.

LBM has a solid theoretical foundation and numerical superiority^8^,^9^,^10^. This simple kinetic theory-based simulation method intrinsically incorporates microscopic and mesoscopic physical mechanisms. The algorithm is simple, easy to program, highly scalable, and convenient in dealing with the complex boundaries of porous media^11^,^12^. Given these features, LBM is particularly suitable for the flow simulation of complex geometries. The feasibility of the LBM method for simulating micro gas flows had been studied^13^. Zhang et al^14^ applied Maxwellian scattering kernel to the wall conditions with an accommodation coefficient. Zhang et al^15^ and Guo et al^16^ discussed the multiple relaxation time method whose relaxation times include *Kn* dependency. Toschi et al^17^ introduced a virtual wall collision concept into the bounce-back and diffuse scattering boundary conditions.

All these previous developments have made the LBM a very competitive technology for shale gas simulations. Here we aim at laying out systematically the methodology and clarifying the possible technical issues that might be encountered with the final goal of building up a solid reliable method for acquiring the flow characteristics of shale gas. In this study, gas flow characteristics under the influences of the slip effect and high Knudsen numbers in organic nano-pores capillaries are simulated with LBM. Gas flow is confined in the tubes with flow direction from left to right, as shown in Fig. 1. The width of the capillary significantly affects simulation results and is thus discussed separately as a sensitivity factor. Mixed boundary conditions that produce the slip effect at pore scales are also discussed. At last, permeability equation which is added a coefficient is provided to model the effect of high Knudsen number and slip.

## Results

### Relaxation time at high Knudsen numbers

**Figure 2:**
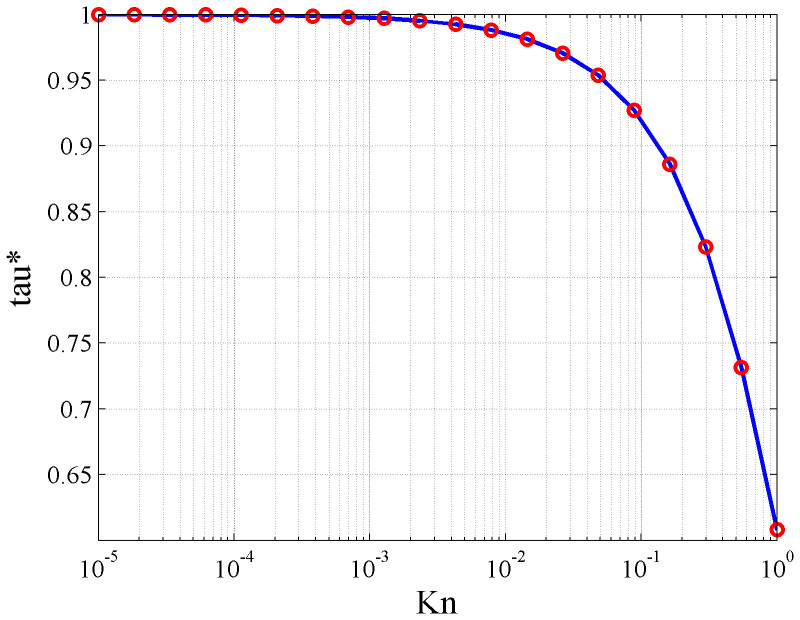
Relationship between relaxation time and Knudsen number.

Reynolds number, *Re*, which is related to fluid shear viscosity *η*, is the basic characteristic quantity of fluid flow in macroscopic flow. Relaxation time *τ* can be uniquely determined by the Reynolds number according to the relationship between *τ* and viscosity when LBM simulation is used for macroscopic flow. With continuum flows (*Kn*<10^−3^), τ can be assumed to be constant. In the LBM model, the second-order error due to spatial discretization can be absorbed into the hydrodynamic equation, yielding a kinematic viscosity^18^ as follows: 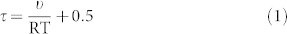
where *υ* is the kinematic viscosity, R is the ideal gas constant, *T* is the temperature. In D2Q9 model, RT is usually taken as 1/3. The Knudsen number *Kn* is the characteristic parameter for nano-scale shale gas flow in porous media, i.e., in the case of slip flow (10^−3^<*Kn*<10^−1^). Thus, one key issue of the LBM simulation of micro-scale flow is to give the right relaxation time for a specified *Kn*. According to kinetic theory, the molecular mean-free-path of a gas and the Knudsen number^19^ can be calculated according to Eq. (2) and (3)^20^. 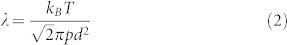

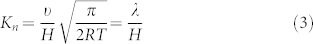
where *λ* is the molecular mean-free-path; *k_B_* is the Boltzmann constant; *d* is the molecular diameter; *p* is the fluid pressure; *T* is the ambient temperature; *υ* is the kinematic viscosity; *H* is characteristic length of flow. Relaxation time *τ* should be corrected according to the Knudsen number *Kn*. Correction is calculated by Eq. (4) and Eq. (5)^13^: 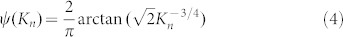

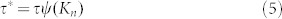
where the modified relaxation time τ* introduces the effect of the pore walls. In the continuum flow region, relaxation time remains substantially constant. However, in the slip flow regime, relaxation time decreases when the Knudsen number increases as shown in Fig. 2, indicating that the collision portion of the actual particles has a short relaxation time, and slip velocity exists on pore walls in the macro-level.

### Boundary conditions

In this study, periodic boundary condition is used for inlet and outlet boundaries. This boundary ensures exact conservations of the mass and momentum of the entire system and provides the best stability compared with boundary conditions with a given pressure or speed.

Two types of boundary conditions for solid wall, namely, the bounce-back boundary condition and the specular-reflection boundary condition, are widely used for top and bottom boundaries. On the nanometer scale, the gas velocity has a slip effect on the boundary. For continuum flow with *Kn* trending to zero, no slip velocity is observed on the wall. However, when *Kn* is finite, the slip velocity is no longer zero. Succi^21^ has used a combination of the bounce back and specular reflection to generate the slip effect at the wall. For shale gas with slip effect, we also use this mixed format to reflect slip effect, 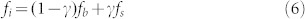
where *f*_b_ is the bounce-back portion, *f*_s_ is the specular-reflection portion, and *γ* is the combination coefficient. *γ* = 0 indicates that the mixed format degenerates to bounce-back. When *γ* = 1, the degradation is specular-reflection and thus theoretically corresponds to the full slip boundary. Therefore, *γ* is used to represent the degree of slip effect.

### Algorithm verification

**Figure 3:**
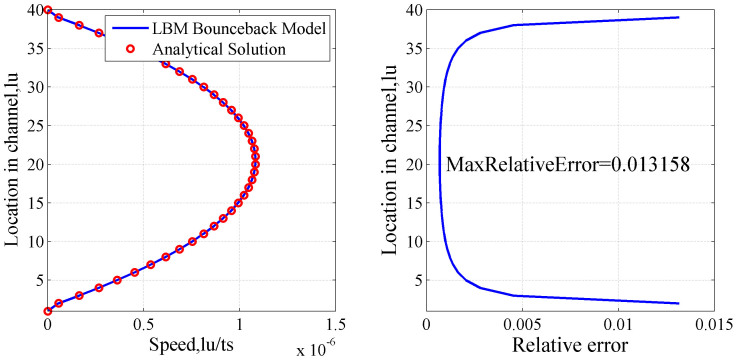
Comparisons (a) and relative errors (b) between analytical solutions and LBM simulation results.

LBM simulation results are compared with theoretical solutions based on classic Poiseuille's law to verify the reliability of LBM simulation. The relative errors of the LBM simulation results with the analytical solutions are also calculated. System temperature *T* is fixed to ambient temperature (298.15 K/25°C); capillary pressure *p* is set to 10 MPa; capillary width is 1 μm; and capillary length *L* is 10 μm when the lattice unit is 25 nm. The fluid is pure methane gas with density of 0.076 g/cm^3^, and the diameter *d* of the gas molecules is 3.8 × 10^−10^ m. The relaxation time of the continuum flow region is 1. The speed driven is used, and the speed increment along the *x* axis Δ*U* is 1 × 10^−9^ m/s. We assume that the change of density and temperature in the channel is small enough that the Knudsen number *Kn* remains almost constant along the entire length of the capillary. For consistency with Poiseuille's theory, the bounce-back format with no-slip boundary condition is used. According to the above set of parameters of the capillary and flow fluid, the Knudsen number is calculated as 6.4 × 10^−4^, and flow is in the continuum region. Comparisons and relative errors between the LBM simulation results and analytical solutions are shown in Fig. 3.

As shown in Fig. 3, the flow velocities are zero on the capillary walls and reach a maximum value at the center point. The LBM simulation results (solid line) and Poiseuille's flow analytical solutions (hollow circles) have good consistency, thus verifying the reliability of the numerical simulation of LBM. These comparisons provide a theoretical foundation for the further use of LBM to study flow characteristics in complex porous media.

## Discussion

### Flow ability for different pore scales

**Figure 4:**
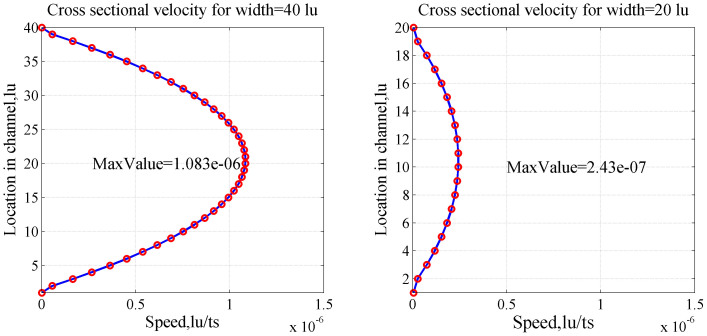
Cross-sectional velocity profiles U_X_ for 40 (a) and 20 lattice width units (b).

One inherent characteristic of shale gas is its nano-scale pores. To study the flow characteristics of shale gas, the width of the capillary model is changed to characterize the change of pore scale. The effect of pore scale on gas flow is studied. Two values, namely, 1 μm and 20 nm, are set as the widths of the capillary model. LBM simulation is used to study the distribution of flow velocity under two different pore scales without considering a high Knudsen number and the slip effect. Fig. 4 shows the simulation results.

On the basis of Poiseuille's law, the average speed of the capillary with 40 (H1) and 20 (H2) lattice units should follow the following equation: 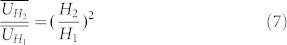
Thus, the ratio of the average velocity is equal to the ratio of the square of the width of the capillary. According to Fig. 4(a), the predicted flow velocity in the capillary with 40 lattice units is between 0 and 1.083 × 10^−6^ lu/ts. According to Eq. (7), the maximum speed of the tube should be 1.083 × 10^−6^ × (20−2)^2^/(40−2)^2^ = 2.43 × 10^−7^ (minus 2 refers to the two lattice units of the tube walls) when the lattice units are reduced to 20. The LBM simulation results show agreement with the calculated values in Fig. 4(b), indicating that the flow rate decreases because of the decrease of capillary width and permeability. The ratio of absolute permeability is equal to the ratio of the square of the capillary width, following classical Darcy's law.

### High Knudsen number

The relaxation time used in the simulation is related to the Knudsen number. When the capillary width decreases, the Knudsen number increases, and the relaxation time should be corrected. Capillary length is fixed on 200 nm, and the width is changed to study the effect of the Knudsen number.

**Figure 5:**
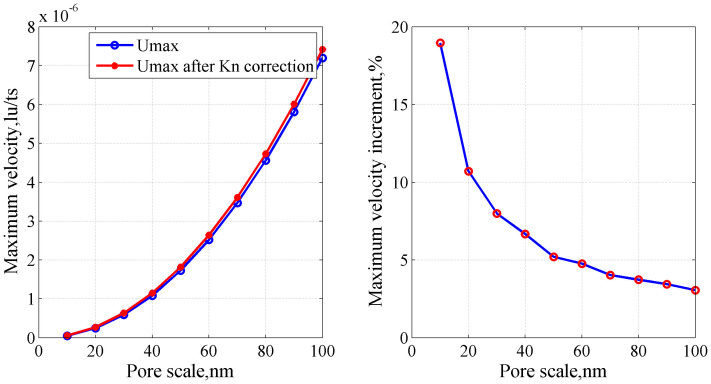
Maximum velocity (a) and maximum velocity increment (b) with width.

Fig. 5 shows that the maximum velocity increases with increasing width. After the relaxation time is corrected with the Knudsen number, velocity increases, and the increment of the velocity becomes more significant when the width decreases. These results suggest that when the pore scale decreases, the correction of relaxation time with the Knudsen number becomes more significant.

### Slip effect

**Figure 6:**
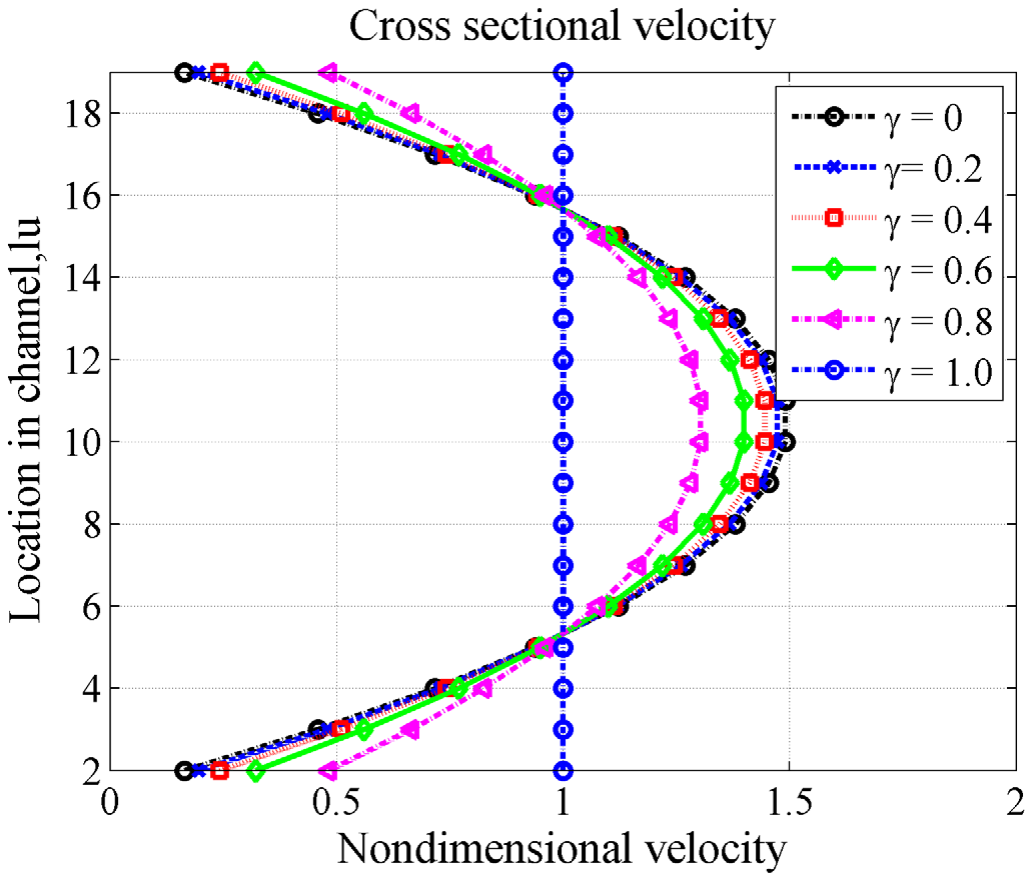
Cross-sectional velocity profile U_X_ with width 20 nm (Kn = 3.2 × 10^−2^) for different slip coefficients.

For gas flow in the nano-scale porous media of gas-bearing shale, the Knudsen number increases to the slip flow region. The capillary with a width of 20 nm remains as the research object to discuss flow characteristics with the slip effect. Mixed boundary condition BSR is adopted to study the extent of the slip effect with the change of the combination coefficient *γ*, such as in Eq. (6). The combination coefficient *γ* determines the slippage effect in the tube velocity. A significant role of the slip effect is that gas flow velocity on the wall is not zero (see Fig. 6).

As shown in Fig. 6, the slip effect significantly increases the flow rate in the tube. The higher the coefficient *γ* is, the higher the velocity. In addition, the velocity profile of the tube changes from the parabolic to a flat shape for the total slip boundary. This finding indicates that at this time, the flow mechanism no longer follows Poiseuille's law and classical flow theory cannot be used to calculate the flow velocity profile and permeability.

**Figure 7:**
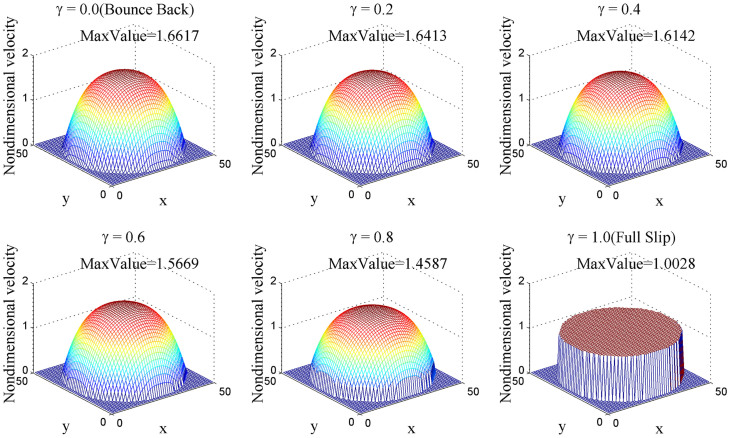
Velocity distribution profile in 3D simulation.

We also performed 3D numerical simulations to represent this phenomenon as shown in Fig. 7. For a tube whose diameter is 50 and length is 200, we cut a cross profile perpendicular to z direction which is along the flow direction. Results are generally the same as 2D simulations.

### Permeability equation

**Figure 8:**
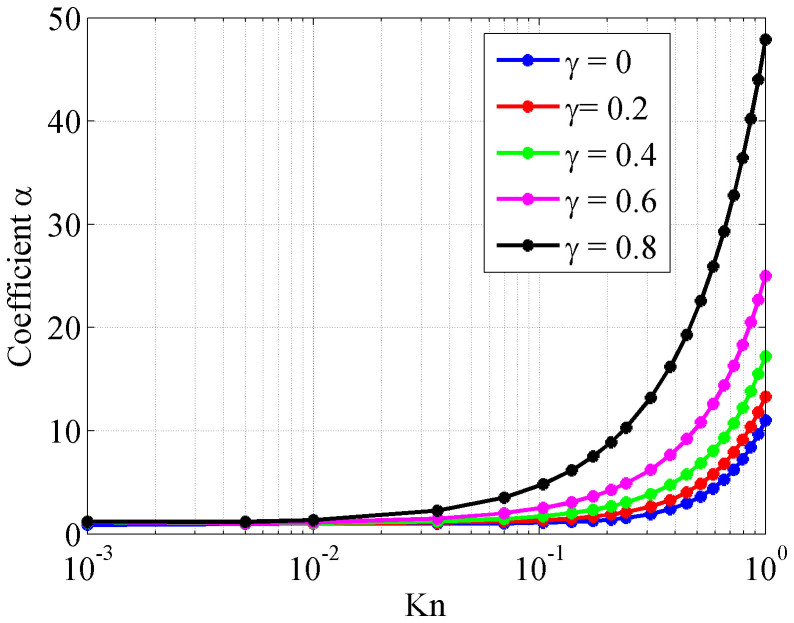
Relationship between coefficient and Knudsen number.

In continuum flows where *Kn* is smaller than 0.1, permeability is a function of tube radius only. When the pore scale gets smaller which leads to the increase of *Kn*, a coefficient *α* should be added to represent the influence of *Kn*. As shown in Fig. 8, when *Kn* is bigger than 0.1, this coefficient changes significantly and yields higher actual permeability than the value calculated by Poiseuille's Law. Slip effect also plays a significantly positive role in flow ability. For the equation of absolute permeability, we add a coefficient *α* to represent the effect of slip and high Knudsen number as Eq. (8) 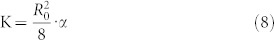
where *K* is absolute permeability of the tube, *R_0_* is the tube radius, *α* is the coefficient to add the effect of high Knudsen number and slip flow.

In this study, LBM is used to simulate the gas flow characteristics of an organic nano-scale capillary, and the Knudsen number and slip effect of shale gas are discussed. With constraints of continuum flow, LBM numerical results are first verified satisfactorily by using analytic solutions according to Poiseuille's law. The effect of different pore scales on the flow rate is discussed. The flow rate is shown to be proportional to the square of the capillary width without a high Knudsen number and slip effect. When the capillary width decreases from the micron scale to the nanometer scale, the Knudsen number increases, the relaxation time should be corrected according to the Knudsen number, and velocity increases. The slip effect significantly increases the capillary flow rate and absolute permeability. The simulation results indicate that LBM provides an effective way to determine shale permeability, which is difficult to experimentally determine. The future trend of development is to apply LBM to three-dimensional porous media network models of digital rocks and predict their absolute permeability to guide shale gas production.

## Methods

### Lattice Boltzmann method

Lattice Boltzmann method is a class of mesoscopic approaches to simulate fluid flows. It is becoming a serious alternative to traditional methods for computational fluid dynamics. Historically, the Lattice Boltzmann approach was developed from lattice gases, although it can also be derived directly from the simplified Boltzmann BGK equation^22^.

In lattice gases, particles live on the nodes of a discrete lattice and jump from one lattice node to the next according to their (discrete) velocities. This is known as the propagation phase. Then, the particles collide with each other and get new velocities, which is known as the collision phase. The simulation proceeds in an alternation between particle propagations and collisions.

The essential ingredients of the LBM are the lattice Boltzmann equation, a space-filling lattice, and a local equilibrium distribution function. The general form of the lattice Boltzmann equation is 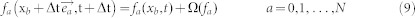
where *f_a_* is the distribution function of particles that travels with velocity 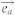. Ω(*f_a_*) is the collision operator which describes changes in particle distribution due to particle collision.

To get correct flow equations it is necessary to use specific lattices that provide sufficient symmetry. The forms of such lattices depend on the dimension of the space. In two dimensions, examples are the square and hexagonal lattices^23^. The 9 speed square lattice known as D2Q9 has been used extensively, where each particle moves one lattice unit at its velocity defined by 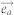 (as Eq. (10)) and in one of the eight directions indicated with 1–8. Particle at position 0 is called the rest particle that has a zero velocity. 
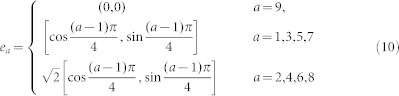
The Bhatnagar-Gross-Krook (BGK) collision operator with a single relaxation time is often used. The BGK collision operator is derived based on linearization of the collision operator around the equilibrium state, neglecting the higher-order terms, and assuming Ω*_a_* (*f_eq_*) equal to zero. Therefore, the BGK collision operator can be written as Eq. (11)^24^, 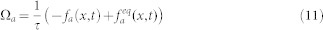
where *τ* is the relaxation time, 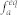 is the local equilibrium distribution function which defines what type of flow equations are solved using the lattice Boltzmann equation. 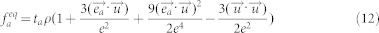
where the weights t_a_ are equal to t_9_ = 4/9, t_1_ = t_3_ = t_5_ = t_7_ = 1/9, t_2_ = t_4_ = t_6_ = t_8_ = 1/36 and 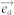 is lattice velocity defined as the lattice size (*Δx*) over the lattice time step (*Δt*). Fluid density (ρ) and velocity (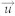) are macroscopic quantities that can be obtained as Eq. (13) and Eq. (14), 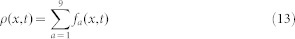

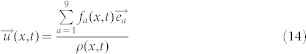


## Author Contributions

X.L.Z. wrote the main manuscript text and prepared figures 1–8. All authors reviewed the manuscript. L.Z.X. and X.W.S. supervised the theoretical analysis and writing. L.G. helped to do the 3D simulation and prepare the data sets of figures 7–8.

## Figures and Tables

**Figure 1:**
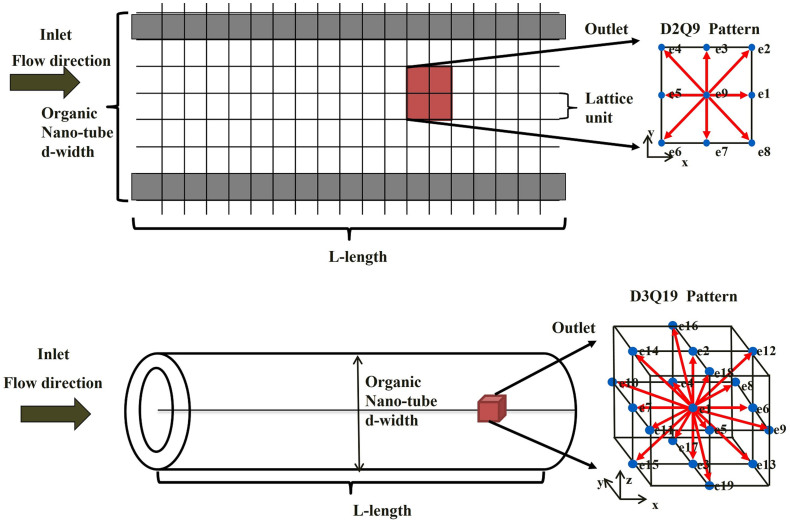
Lattice unit and flow direction of 2D and 3D organic capillaries.
